# Gypenoside-14 Reduces Depression via Downregulation of the Nuclear Factor Kappa B (NF-kB) Signaling Pathway on the Lipopolysaccharide (LPS)-Induced Depression Model

**DOI:** 10.3390/ph16081152

**Published:** 2023-08-14

**Authors:** Yaqun Jiang, Xiang Cheng, Ming Zhao, Tong Zhao, Mengya Zhang, Zibi Shi, Xiangpei Yue, Yanan Geng, Jiayue Gao, Chengbo Wang, Junli Yang, Lingling Zhu

**Affiliations:** 1 School of Pharmaceutical Sciences, University of South China, Hengyang 421001, China; yaqunjiang2023@163.com (Y.J.); zhmyasz@163.com (M.Z.); 2 Beijing Institute of Basic Medical Sciences, Beijing 100850, China; mac_chx@163.com (X.C.); mingzhao28081@gmail.com (M.Z.); 13661091893@163.com (T.Z.); zibishi1994@163.com (Z.S.); xiangpei2015@sina.com (X.Y.); gengtangrong@163.com (Y.G.); gaojy0228@163.com (J.G.); 3 CAS Key Laboratory of Chemistry of Northwestern Plant Resources and Key Laboratory for Natural Medicine of Gansu Province, Lanzhou Institute of Chemical Physics (LICP), Chinese Academy of Sciences (CAS), Lanzhou 730000, China; wangcb@licp.cas.cn; 4 Co-Innovation Center of Neuroregeneration, Nantong University, Nantong 226019, China

**Keywords:** gypenoside-14 (GP-14), depression, lipopolysaccharide, astrocyte, neuroinflammation

## Abstract

Neuroinflammation is a common pathogenetic sign of depression and is closely linked to the development of depression. Many clinical anti-inflammatory drugs act as antidepressants by reducing the neuroinflammatory response. Previous research found that gypenosides and their bioactive compound gypenoside-14 (GP-14) had neuroprotective effects against hypoxia-induced injury and reduced neuroinflammation-related high-altitude cerebral edema. Here we investigated the effects of GP-14 on the lipopolysaccharide (LPS)-induced depression-like behavior model. LPS (0.5 mg/kg) was injected into mice intraperitoneally for 7 consecutive days to induce depression-like behavior, which is considered a model for the exacerbation of depression. GP-14 in the amount of 100 mg/kg was simultaneously administered by gavage for 7 days. In the LPS-induced depression model, GP-14 not only attenuated depression-like behavior but also improved the anxiety-like behavior of the mice. Additionally, GP-14 treatment mitigated learning and cognitive decline in depressed mice. ELISA and immunofluorescence staining results revealed that GP-14 inhibited the upregulation of pro-inflammatory cytokines, including tumor necrosis factor-α (TNF-α), interleukin-1β (IL-1β), and interleukin-6 (IL-6), and suppressed the activation of astrocytes induced with LPS, indicating its potent anti-inflammatory effect. GP-14 pretreatment in C8 cells and primary astrocytes can inhibit the activation of the NF-κB signaling pathway and downregulate the levels of pro-inflammatory factors. In summary, our findings showed that GP-14 had significant anti-inflammation and anti-depression properties; thus, GP-14 could be a promising lead compound for treating depression.

## 1. Introduction

Depressive disorder is a prevalent and severe mental illness, which is caused by a combination of environmental, genetic, and epigenetic factors. However, its underlying mechanisms are complex and have not been elucidated, making its clinical diagnosis and treatment difficult [1]. Previous studies have suggested the following hypotheses for the pathogenesis of depression: imbalances in monoamine neurotransmitters, dysfunction in the hypothalamic pituitary adrenal axis (HPA), and an insufficiency of neurotrophic factors [2,3,4]. Among other potential biological factors, the role of neuroinflammation in depression has received increasing attention, and an increasing number of studies have found neuroinflammation to be important in the development of depression [5,6]. Clinical studies have found that patients treated with interferon-α (IFN-α) for hepatitis C exhibit symptoms of depression, also suggesting that IFN-α injections can induce depression [6]. Animal studies also revealed that LPS can significantly increase levels of inflammatory mediators in serum and brain from mice with the depression-like condition [7], whereas treatments with some antidepressant drugs downregulated the levels of these pro-inflammatory cytokines and produced antidepressant-like effects [8,9]. Neuroinflammatory responses, as indicated by these findings, may have a significant role to play in the development of depression. As a result, focusing on inflammation-induced pathogenesis of depression progression has the potential to affect novel therapeutic and preventative approaches to treating this disorder.

The plant *Gynostemma pentaphyllum* Makino is a member of the genus *Gynostemma* of the family Cucurbitaceae. It is extensively employed in Asian countries as a functional food and tea, particularly in traditional medicine. The primary bioactive component of *G. pentaphyllum* is gypenosides (GPs), which exhibit diverse biological properties such as antioxidant, anti-inflammatory, anti-apoptotic, and anti-fatigue [10,11]. Furthermore, GPs played an important role in neuroprotection. For example, the study showed that GPs can induce neurogenesis and alleviate brain injury in stroke [11]. It has been demonstrated that GPs exerted antidepressant-like effects by inhibiting neuroinflammation in mice. GP administration revealed a significant reduction of anxiety-like behavior caused with LPS and a significant reduction in levels of pro-inflammatory mediators in the brain [12]. By inhibiting the activation of microglia and reducing the level of inflammatory factors, GPs improved depression-like behavior in a chronic and unpredictable mild stress model (CUMS) [13]. Gypensapogenin V extracted from *G. pentaphyllum* saponin were able to protect damaged myocardial cells [14]. Gypenoside IX has been found to be able to reduce the inflammatory response of astrocytes [15].

Our previous studies found that GPs had a protective effect on PC12 cells against hypoxia injury and improved spatial memory impairment in mice that had been exposed to hypoxia [16]. Recently, one of the newly identified bioactive components of GPs, GP-14, was found to inhibit microglial activation, suppress the NF-κB pathway, and prevent pro-inflammatory cytokines from being produced in a mouse high-altitude cerebral edema (HACE) model [17]. Additionally, GP-14 pretreatment attenuated neuronal injury under hypoxic conditions through the AKT and ERK pathways [18].

Taken together, based on the literatures and our previous data, in the current study, we investigated whether GP-14 has protective effects on neuroinflammation related-depression. Therefore, we used the LPS-induced depression mouse model to detect the effects of GP-14 on the depression behavior; then we assayed the activation of NF-κB pathway and the expression levels of IL-1β, IL-6, and TNF-α in the serum and hippocampus; finally, the possible mechanisms of GP-14 were investigated in C8 cell lines and primary astrocytes.

## 2. Results

### 2.1. GP-14 Reversed Depressive-like Behaviors Induced with LPS

**Figure 1:**
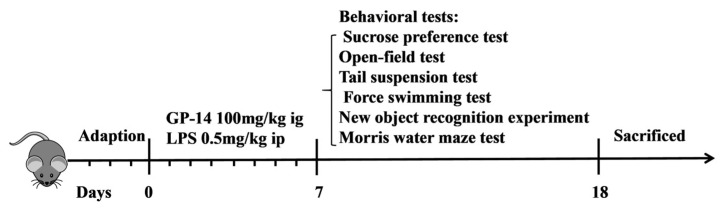
Diagram of the behavioral experimental design.

**Figure 2:**
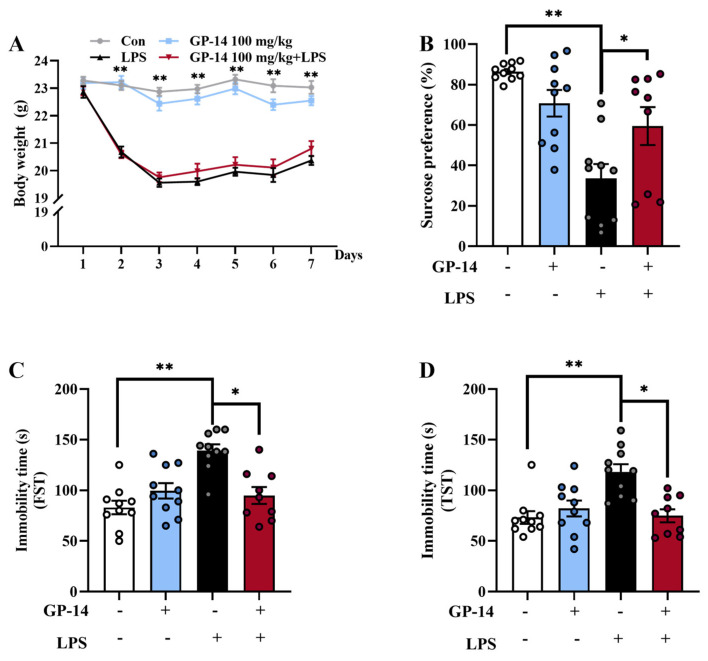
The effect of GP-14 depressive-like behavior in LPS induced-depression. (**A**) The change of body weight. (**B**) Sucrose preference test (SPT). (**C**) Immobility time in FST. (**D**) Immobility time in TST. The data are presented as mean ± SEM (*n* = 9−10/group). * *p* < 0.05, ** *p* < 0.01.

In our previous studies, GP-14 of 100 mg/kg and 200 mg/kg were used to treat LPS-induced high-altitude cerebral edema. GP-14 at 100 mg/kg significantly alleviated neuroinflammation and blood brain barrier (BBB) disruption in a mouse HACE model [17]. Therefore, the dosage of GP-14 100 mg/kg was selected to conduct the present experiment. Firstly, we analyzed the changes in body weight between groups of mice to determine whether GP-14 might influence depression induced with LPS. Figure 1 shows, mice were treated for 7 days with LPS injection along with GP-14 gavage administration. Compared with the control group, the LPS group mice revealed a significant decrease in the first three days and gradually increased in the following days. However, the mice subjected to LPS induction did not exhibit any significant changes in body weight following the administration of GP-14 at a dosage of 100 mg/kg (Figure 2A).

In order to verify the success of the LPS-induced depression model and investigate the effects of GP-14 on depressive behaviors in the animal model, we evaluated the behaviors of the mice in each group via sucrose preference test (SPT), tail suspension test (TST), and forced swim test (FST). As shown in Figure 2B, the sucrose preference rate was significantly reduced in LPS group mice compared with the control group. However, this trend was reversed with GP-14 treatment. In FST (Figure 2C), LPS mice remained immobile longer than control group mice, but GP-14 treatment reversed this effect. Similarly, TST (Figure 2D) showed a similar trend to FST. These results indicated that LPS induces depression successfully in mice, suggesting that GP-14 can effectively reverse LPS-induced depression-like behavior.

### 2.2. GP-14 Attenuated LPS-Induced Anxiety-like Behavior

**Figure 3:**
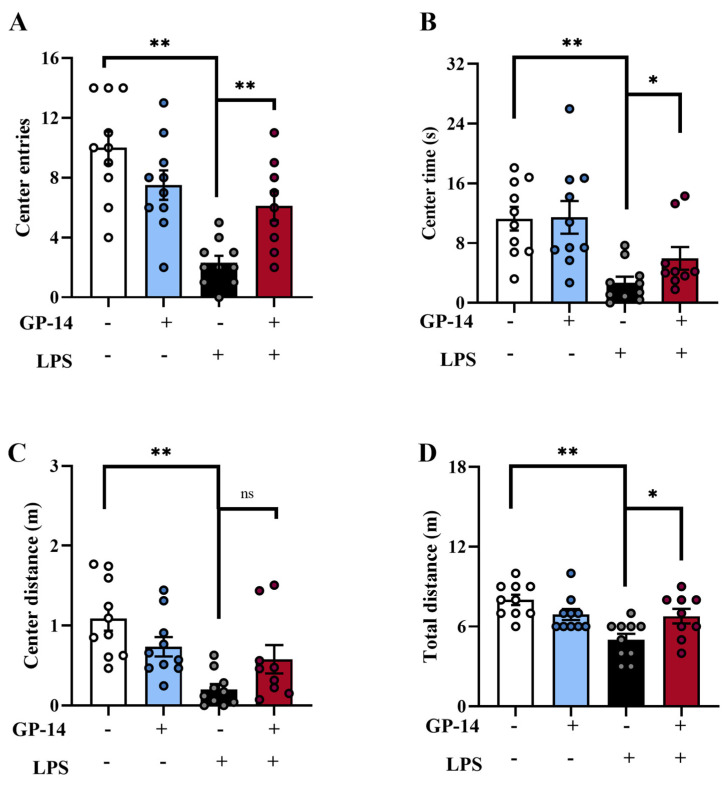
The effect of GP-14 anxiety-like behavior in the LPS-induced model. (**A**) Center entries in the OFT. (**B**) Center time in the OFT. (**C**) Center distance of the central area. (**D**) Total distance in the open field test. The results were presented as mean ± SEM (*n* = 9−10/group). * *p* < 0.05, ** *p* < 0.01. ns means no significant difference.

To determine whether GP-14 affects anxiety-like behaviors, we conducted an evaluation of the anxiety-like behavior of mice with the open field test (OFT). As shown in Figure 3A,B, LPS treatment decreased the entries and time spent in the central area of the open field in mice, but after GP-14 treatment, entries and spent time in the central area of the open field were increased. Meanwhile, compared with the LPS group, the central distance had no difference with GP-14 treatment (Figure 3C), but the total distance was significantly increased (Figure 3D). This result suggested that GP-14 can alleviate anxiety-like behavior in LPS-induced model.

### 2.3. GP-14 Improved Impairments of Learning and Memory Induced with LPS

**Figure 4:**
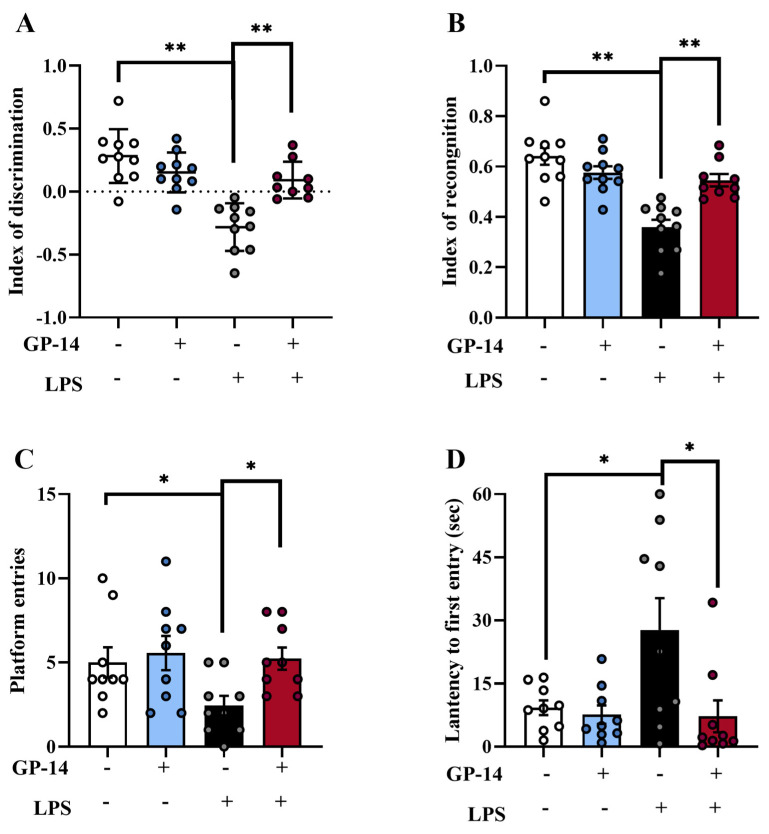
The effect of GP-14 on learning and memory in LPS-induced depression. (**A**) The discrimination index of novel object recognition was detected (*n* = 9−10/group). (**B**) The cognitive index of novel object recognition was evaluated (*n* = 9−10/group). (**C**) The mean number of platform entries was detected during MWM (*n* = 9/group). (**D**) The latency to first entry was recorded with MWM (*n* = 9/group). The results were presented as mean ± SEM. * *p* < 0.05, ** *p* < 0.01.

Depression is considered to be a central nervous system disorder that can impair cognitive function in patients. Therefore, we detected changes in learning and the spatial memory of mice with the new object recognition experiment (NOR) and the Morris water maze test (MWM). Firstly, we examined the change in learning of mice using NOR. In the NOR test, new target exploration time were significantly decreased and the ability to discriminate between old and new objects were reduced in the LPS group, compared to the control group (Figure 4A,B); however, GP-14 treatment can reverse this difference. Subsequently, MWM was used to detect changes in spatial memory in mice. Similarly, in MWM (Figure 4C,D), compared to the control group mice, the spatial memory of mice with LPS treatment was impaired, which embodied as a reduction in the number of successful crossings and short latency. However, the phenomena caused with LPS stimulation were reversed after GP-14 treatment. In conclusion, these results demonstrated that treatment with GP-14 exerted a beneficial effect on LPS-induced learning and memory deficits.

### 2.4. GP-14 Alleviated LPS-Induced Neuroinflammation

**Figure 5:**
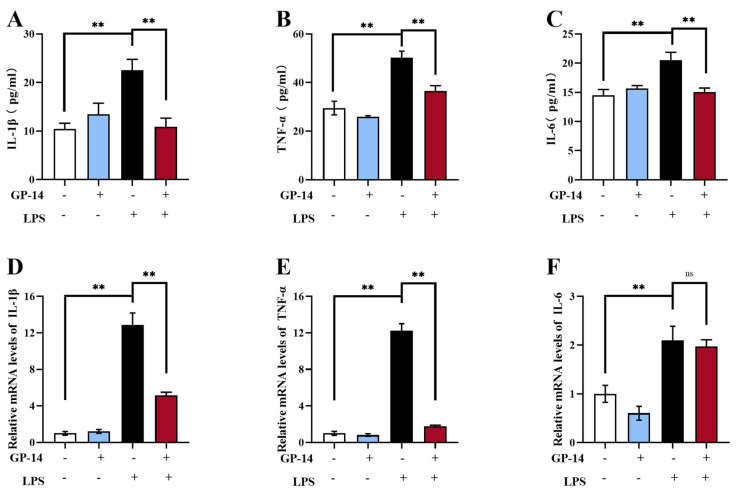
Changes of pro-inflammatory cytokines (IL-6, IL-1β and TNF-α) in LPS-induced mice. (**A**–**C**) The IL-6, IL-1β, and TNF-α levels in serum were measured with ELISA kits. Data are expressed as mean ± SEM (*n* = 8/group). ** *p* < 0.01. (**D**–**F**) QPCR was utilized to detect the mRNA levels of IL-1β, IL-6, and TNF-α in the hippocampus. The results were expressed as mean ± SEM (*n* = 4−5/group). ** *p* < 0.01, ns means no significant difference.

To evaluate the anti-inflammatory effects of GP-14, the levels of inflammatory mediators in the serum and hippocampal were detected using ELISA and Quantitative PCR (qPCR). As shown in Figure 5A–C, GP-14 treatment effectively inhibited the increase of pro-inflammatory cytokines, for example, IL-6, IL-1β, and TNF-α, in the serum from mice in the LPS-induced depression model. The qPCR assay data displayed that, in the hippocampus, GP-14 treatment exhibited the ability to effectively decrease the elevated mRNA levels of IL-1β and TNF-α, which are caused with LPS stimulation, but IL-6 levels were not obviously decreased (Figure 5D–F). Similarly, the ELISA assay data displayed that GP-14 treatment decreased the levels of inflammatory mediators in the hippocampus tissue in the LPS-induced depression model (Figure S1). These data suggest that GP-14 alleviated LPS-induced neuroinflammation.

### 2.5. GP-14 Inhibited LPS-Induced Activation of Astrocytes

**Figure 6:**
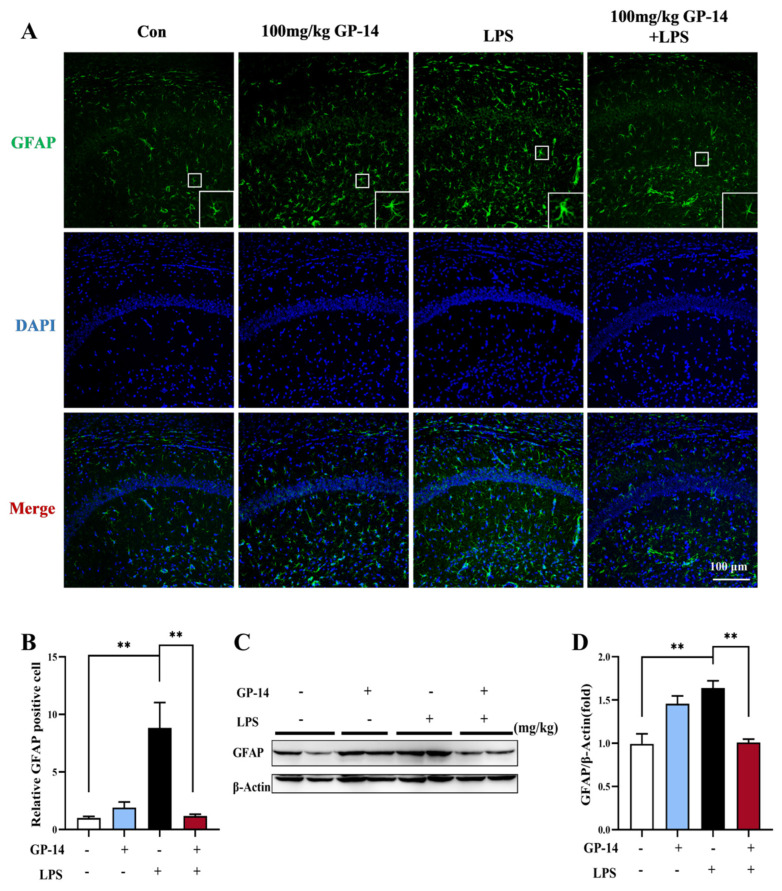
The effect of GP-14 on astrocytes in LPS-induced depression. (**A**,**B**) Representative images and statistical analysis of astrocytes in the CA1 region of the hippocampus. (**C**) The expression of the astrocyte activation marker glial fibrillary acidic protein (GFAP) was assessed with western blotting. (**D**) Measuring the intensity of protein bands corresponding to glial fibrillary acidic protein (GFAP). The results were presented as the mean ± standard error of the mean (SEM) with a sample size of 3−4 per group. Statistical significance was denoted with ** *p* < 0.01. The boxes represented an enlarged single astrocyte.

To examine whether astrocytes are involved in LPS-induced neuroinflammation, the expression of the astrocyte activation marker glial fibrillary acidic protein (GFAP) was assessed with IF staining and western blotting. As shown in Figure 6A,B, we observed that GFAP expression in the CA1 region of the hippocampus was significantly increased after LPS treatment, indicating that astrocytes were damaged. Interestingly, GP-14 treatment markedly inhibited the activation of astrocytes induced with LPS. Similarly, the results of the western blot showed that GFAP expression was upregulated after LPS treatment compared to the control group. However, this trend was resumed by GP-14 (Figure 6C,D). These results further confirmed that GP-14 can inhibit neuroinflammation.

### 2.6. Suppression of Inflammatory Response and NF-κB Pathway Activation with GP-14 in C8 Cells and Primary Astrocytes

**Figure 7:**
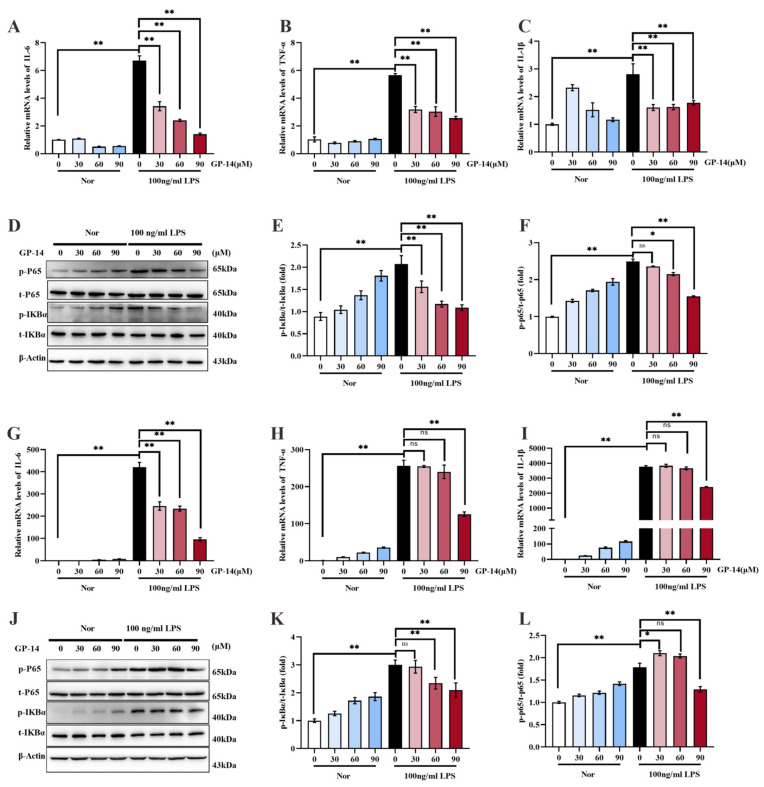
The effect of GP-14 on the NF-κB signaling pathway in C8 cells and primary astrocytes. (**A**–**C**) The C8 cells were subjected to various concentrations of GP-14 for a duration of 6 h. Subsequently, the cells were exposed to 100 ng/mL of lipopolysaccharide (LPS) for another 6 h, with some cells being left untreated. QPCR measured the levels of IL-6, IL-1β, and TNF-α. (**D**) Protein bands of phosphorylated IκBα (p-IκBα), phosphorylated p65 (p-p65), total-IκBα (t-IκBα), total-p65 (t-p65), and β-actin in C8 cells. (**E**,**F**) Quantification of protein bands for phosphorylated-IκBα and phosphorylated-p65 in C8 cells. (**G**–**I**) The primary astrocytes were treated with different doses of GP-14 for 6 h, followed by treatment with or without 100 ng/mL of LPS for 6 h. The levels of IL-6, IL-1β, and TNF-α were measured in primary astrocytes using qPCR. (**J**) Protein bands of phosphorylated IκBα (p-IκBα), total IκBα (t-IκBα), phosphorylatedp65 (p-p65), total-p65 (t-p65), and β-actin in primary astrocytes. (**K**,**L**) Quantification of protein bands of phosphorylated IκBα (p-IκBα) and phosphorylated p65 (p-p65) in primary astrocytes. The results are expressed as the mean ± SEM (*n* = 3), * *p* < 0.05, ** *p* < 0.01. ns means no significant difference.

We detected the role of GP-14 in order to explore the underlying mechanisms for the anti-depressant effect of GP-14 in astrocyte cell line C8. The C8 cells were treated with or without 100 ng/mL of LPS for 6 h after using different doses of GP-14, treated for 6 h. As Figure 7A–C shows, GP-14 suppressed the increase of mRNA levels of three cytokines (such as IL-6, TNF-α, and IL-1β) that were induced with LPS stimulation. The protein expression of both phosphorylated IκB-α (p-IκBα) and phosphorylated p65 (p-p65) was significantly increased with LPS compared to the control group. Pretreatment with 90 μM of GP-14 resulted in a significant reduction of protein expression of both p-IκBα and p-p65. However, the total protein remained unaffected. The findings of this study suggest that GP-14 possessed the capability to effectively inhibit the activation of the NF-κB pathway, which is triggered with LPS (Figure 7D–F). In summary, GP-14 attenuated the inflammatory response in C8 cells through inhibiting the activation of the NF-κB pathway. Subsequently, we investigated the role of GP-14 on neuroinflammation in primary astrocytes. Similarly, 90 μM of GP-14 pretreatment significantly decreased the levels of IL-6, TNF-α, and IL-1β after LPS treatment (Figure 7G–I). The results of western blot analysis also indicated that 90 μM of GP-14 pretreatment significantly inhibited the upregulation of p-p65 and p-IκBα levels that were induced with LPS treatment, suggesting that GP-14 was able to inhibit the NF-κB pathway activation in primary astrocytes (Figure 7J–L). Thus, GP-14 played an anti-inflammatory role in C8 cells and primary astrocytes, which is associated with the NF-κB pathway.

## 3. Discussion

**Figure 8:**
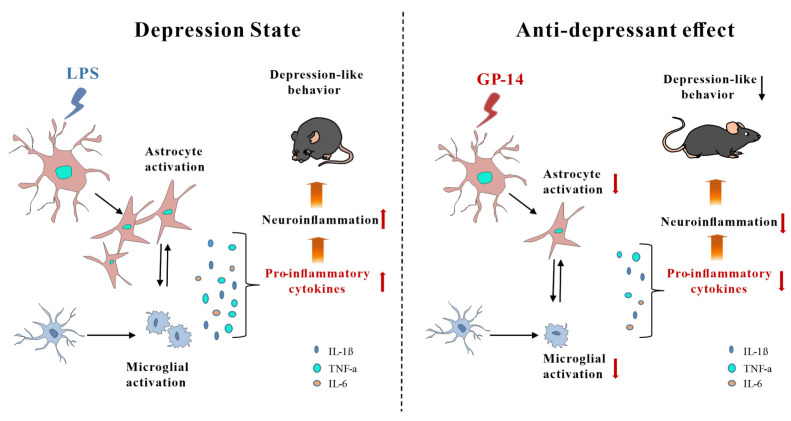
Model showing the role of GP-14 in LPS-induced depression-like behaviors in mice. GP-14 reduces depression-like behavior in mice by inhibiting astrocytes activation and decreases levels of pro-inflammatory factors.

In this study, we demonstrate that GP-14 extracted from gypenosides reduced neuroinflammation by inhibiting astrocyte activation via downregulation of the NF-κB signaling pathway in a LPS-induced mice model and then improved depression-like behavior (as shown in Figure 8). The results showed that GP-14 not only reversed LPS-induced depression-like behavior and reduced anxiety-like behavior but also improved LPS-induced memory impairment in mice. At the molecular level, GP-14 inhibited the activation of the NF-κB signaling pathway and reduced the levels of inflammatory factors. These results indicate for the first time that GP-14 could be a novel therapeutic approach for the treatment of depression.

The pathogenesis of depression is highly complex, and neuroinflammation is considered to play a significant role in the development of psychiatric disorders, including depression [19]. Previous clinical studies have found that depressive behavior is associated with elevated levels of pro-inflammatory cytokines in the central and peripheral nerve [20]. The development and progression of inflammatory diseases are critically influenced by pro-inflammatory cytokines, such as IL-1β, IL-6, and TNF-α [21]. These inflammatory mediators activate microglia and astrocytes in the brain, leading to central nervous system inflammation and synaptic dysfunction, which can lead to depression [22]. Therefore, alleviating inflammation may be an effective means of ameliorating depression.

LPS is a commonly used pro-inflammatory mediator that produces pro-inflammatory cytokines and releases inflammatory mediators in serum and brain [23]. The increased inflammatory factors in animal brains result in evident depressive-like behaviors, including reduced preference for sucrose, longer periods of immobility in the TST and FST, as well as decreased locomotor activity in the OFT [24,25]. In addition, depression can also lead to cognitive impairments [25,26]. To assess the efficacy of antidepressant therapies in animal models of LPS-induced depression, various behavioral assessments such as SPT, FST, and TST are commonly utilized. The FST and TST are utilized as assessments for behavioral despair, offering understanding into the degree of depression in animals by observing their display of hopelessness. SPT is a behavioral indicator used to assess the loss of pleasure or anhedonia. In this study, the decrease of sucrose preference and the increase of immobility time during the TST and FST were considered as indicators of depressive-like behavior. Our research findings indicated that LPS treatment reduced sucrose preference and increased immobility time during the TST and FST in mice. However, the administration of GP-14 significantly improved the depressive state of the mice. Similarly, in the OFT, LPS treatment significantly reduced the total distance traveled and the number of entries into the central area, indicating that the interest in exploring the novel environment decreased and anxiety-like behavior appeared. The NOR and MWM are used to assess the cognitive and memory abilities of experimental animals [27]. In this experiment, LPS treatment resulted in spatial and memory deficits, while GP-14 treatment reversed this phenomenon. In conclusion, these behavioral results demonstrated that GP-14 exhibited antidepressant effects in the LPS-induced mouse model of depression.

Astrocytes can respond to inflammatory signals, promote inflammation, and participate in the regulation of multiple life processes in the nervous system under both physiological and pathological conditions. Reactive astrogliosis is a common neuroinflammatory hallmark in central nervous system disorders. We found that GP-14 can inhibit the activation of astrocytes, suppress the phosphorylation of P65 and IκBα, and prevent the production of TNF-α, IL-6, and IL-1β, both in vivo and in vitro, under inflammatory conditions. During the inflammatory process, the nuclear translocation of the NF-κB heterodimer is a crucial step for astrocyte activation [28,29]. The NF-κB complex is located in the cytoplasm in an inactive condition [30,31]. The nuclear translocation of NF-κB in astrocytes is triggered with pro-inflammatory stimuli, such as TNF-α, IL-1β, and other inflammatory mediators [32]. Once phosphorylated, the NF-κB complex translocates to the cell nucleus, activating the transcription of downstream pro-inflammatory molecules. Our study found that GP-14 can inhibit NF-κB nuclear translocation and transcriptional activity and downregulate the expression of p-p65 and P-IκBα in C8 cells and primary astrocytes. These findings suggest that GP-14 exerts an antidepressant effect by suppressing the activation of astrocytes through the NF-κB pathway.

Recent studies have demonstrated that GP-14 suppressed the NF-κB pathway in microglia and blocked the generation of pro-inflammatory factors to reduce plateau brain edema [17]. Our study found that GP-14 can reduce the production of inflammatory mediators induced with LPS in the hippocampus. However, the anti-inflammatory and antidepressant effects of GP-14 may be a collective response of central nervous system glial cells. While GP-14 can inhibit the activation of astrocytes in the mouse, it does not rule out the possibility of direct or indirect inhibitory effects on other neural cells. We need further investigation to explore whether GP-14 can reduce the inflammatory responses of other central nervous system cells besides astrocytes.

The present study also has several limitations. One is that we conducted multiple behavioral experiments in the same cohort of mice. While we have considered and controlled for confounding factors such as time, location, and order, we cannot completely rule out the possibility of interactions between different behavioral tasks. Second, in that article, only male mice were used for the experiments, and no sex differences were taken into account. In our further studies, more detailed investigations should be conducted.

## 4. Materials and Methods

### 4.1. Animals and Treatment

The C57BL/6J mice (male, 8 weeks of age) were purchased from the Laboratory Animal Center of Vital River Experimental Animal Company (Beijing, China). The animals were maintained under controlled conditions (12 h light/12 h dark cycle; temperature, 23 ± 2 °C; relative humidity, 50%–60%), which included free access to standard rodent chow and water. After 3 days of acclimatization, animals were randomly divided into four groups: control group (*n* = 10), LPS group (*n* = 10), GP-14 group (*n* = 10), and GP-14 + LPS group (*n* = 9). GP-14 was provided by the Lanzhou Institute of Chemical Physics (LICP). The dose of LPS was chosen according to the method reported previously [33]. GP-14 was suspended in a 0.5% sodium carboxymethyl cellulose solution. GP-14 was administered via intragastric gavage daily. The control group was given 0.5% sodium carboxymethyl cellulose solution via gavage daily. Mice were gavaged every day at 8:00 a.m. and were intraperitoneally injected with 0.5 mg/kg LPS (Cat. no. L2630, Sigma-Aldrich, St. Louis, MO, USA) at 4:00 p.m. Behavioral tests were performed on the same group of mice 24 h after the last LPS injection. After being perfused with chilled sterile PBS, the brains were extracted cautiously, and serum and hippocampus samples were promptly gathered and preserved at −80 °C until required for subsequent experimentation. Diagram of the behavioral experimental design is shown in Figure 1.

### 4.2. Behavioral Tests

#### 4.2.1. Sucrose Preference Test (SPT)

A reduction in sucrose intake can be used to assess anhedonia in mice. The sucrose preference test was conducted as described in the previous laboratory protocol [34]. Firstly, mice were trained to consume a 1% sucrose solution from two different bottles for 24 h. Later, one of the bottles was changed to pure water for 24 h. To avoid bottle side preference, the two bottles were switched after 12 h. After acclimation, the mice were deprived of water for 12 h, allowing each mouse free access to 1% sucrose and pure water from two different bottles. After 12 h, the quantity in both bottles was measured and sucrose preference was calculated according to the following formula: Sucrose preference (%) = sucrose water consumption/(sucrose + purified water) consumption × 100%. The observer was blind to the treatment and the group of the animal being tested.

#### 4.2.2. Open Field Test (OFT)

The experimental procedure was conducted as previously described [34]. For pre-acclimation, the mice were kept in the test box for 4 h beforehand. In the experiment, each mouse was gently placed into a 50 × 50 × 39 test box. The activity of each mouse in the test box for 5 min was recorded with ANY-maze software.

#### 4.2.3. Tail Suspension Test (TST)

We adopted the previous protocol from laboratory and made modifications based on it [34]. The tails of the mice were taped with medical tape, and mice were suspended 2–3 cm above the ground with their head upside down. The experiment was conducted for 6 min, and the immobility of each mouse was recorded in the last 4 min.

#### 4.2.4. Force Swimming Test (FST)

We adopted the previous protocol from laboratory and made modifications based on it [34]. The forced swimming test was carried out in a glass cylinder with a diameter of 10 cm and a height of 25 cm. The experiment was conducted for 6 min, and the immobility of each mouse was recorded in the last 4 min.

#### 4.2.5. New Object Recognition Experiment (NOR)

According to the method described previously [34], the new object experiment was divided into three days: adaptation, familiarization, and testing. In the first day, mice were given 5 min to adapt to the environment under a “no objects” condition. In the second day, there were two identical objects A in the recognition device, and the mice were placed with the recognition device for 10 min to familiarize themselves with objects A. During the last day, one object A in the recognition apparatus was replaced with object B. The experiment was conducted for a total of 15 min, and the time taken by the mice to recognize the object during the next 10 min was recorded. To assess the recognition of new objects, the recognition index (RI) was calculated using the formulas RI = N − F/(N + F) and the discrimination index DI = N/(N + F), where N represents the total time that the mouse’s nose was in contact with object B and F represents the total time that the mouse’s nose was in contact with object A.

#### 4.2.6. Morris Water Maze Test (MWM)

We adopted the previous protocol from laboratory and made modifications based on it [34].The water maze used in this study consisted of a circular tank with a diameter of 160 cm and a platform filled with tap water at a temperature of 23 ± 2 °C. The swimming trajectory in the water maze was automatically recorded by a computer. In acquisition trials lasting 5 days, the platform was submerged 2 cm below the water surface, and mice were placed in the maze in one of the four quadrants facing the curtain of the tank. Mice were allowed to search the platform for 60 s. Once the mice failed to find the platform, they were guided to stay on the platform for 10 s. Four trials were performed each day with at least 1 h between trials. Escape latency was recorded for each trial, which indicated the acquisition of spatial memory. On day 6, the platform was removed, the probe test was performed, and the mice were placed in the water in the quadrant farthest from the hidden platform. Each mouse was allowed to swim in the water for 60 s. During this time, the time taken by the mice to find the platform for the first time and the number of times they passed through the platform were recorded.

### 4.3. Biochemical Assessment

#### 4.3.1. C8 Cell Culture and Treatments

C8 cells were obtained from the Cell Resource Center of the Chinese Academy of Medical Sciences (Beijing, China). DMEM was used to culture the C8 cells, which contained 10% (*v*/*v*) fetal bovine serum (FBS, Gibco, Waltham, MA, USA). After seeding cells in cell culture plates, C8 cells were pretreated with 30, 60, and 90 μM GP-14 for a duration of 6 h. Subsequently, the cells were treated with LPS for another 6 h. Following cell collection, qPCR and western blotting were performed.

#### 4.3.2. Primary Astrocytes Culture and Treatments

Neonatal mice on postnatal day 1 were used to obtain primary astrocytes from their cortices. In short, the cortex was meticulously separated from the entire brain and subsequently finely diced into small fragments. The cells underwent digestion using 0.125% trypsin in a cell incubator set at 37 °C for a duration of 30 min. Next, the cells were introduced into tissue culture flasks of 75 cm^2^ using DMEM/F12 enriched with 10% FBS and penicillin/streptomycin (100 units and 100 µg/mL). Fresh DMEM/F12 medium was replaced every 3 days after a 24-h period. Following a week, the cells that were mixed underwent vigorous shaking throughout 30 min, and subsequently, the liquid above was eliminated. Cell culture plates were used to seed cells, and primary astrocytes were pretreated with GP-14 at concentrations of 30, 60, and 90 μM for a duration of 6 h. Subsequently, the cells were treated with LPS for 6 h. After collecting the cells, qPCR and western blotting were conducted.

#### 4.3.3. Immunofluorescence

The mice were heavily sedated using 1% sodium pentobarbital and subsequently flushed with (0.9%) saline solution in order to eliminate circulating blood cells. The brain was immersed in 4% PFA at a temperature of 4 degrees Celsius for a duration of 24 h. Subsequently, it was moved to a solution containing 15% sucrose for an additional 24 h. Finally, the brain underwent dehydration in a solution of 30% sucrose at a temperature of 4 degrees Celsius for a period of 48 h. Following the saturation of sucrose, the brain underwent slicing into sections that were 40-μm-thick using a frozen sectioning machine (Thermo model E, located in Waltham, MA, USA). The sections that included the hippocampal area underwent permeabilization using 0.5% Triton X-100 and were then blocked with 5% BSA at a temperature of 37 °C for a duration of 30 min. GFAP (Cat. no. ab279302, Abcam, Waltham, MA, USA; dilution 1:500) was incubated with sections overnight at 4 °C. After being washed three times (10–12 min) in a PBS solution, the sections were then treated with anti-rabbit secondary antibodies Alexa Fluor 488 for 60 min at room temperature. The cell nuclei were stained again using a mounting medium that contained DAPI (Cat. no. ZLI-9557, ZSGB-BIO, Beijing, China). Acquisition of the images was performed with a scanning confocal microscope of high resolution (Nikon, Tokyo, Japan).

#### 4.3.4. Western Blotting

Hippocampus and cultured astrocytes were homogenized using RIPA lysis buffer on ice. Each sample was separated with 10% SDS-PAGE gels and transferred to PVDF membrane, which was blocked with 5% nonfat milk at room temperature for 1 h. After washing four times with Tris-buffered saline containing Tween 20, the membrane was incubated with the indicated primary antibodies at 4 °C with shaking overnight. The p-IκBα, t-IκB-α, p-p65, t-p65, and β-actin were incubated with the same membrane which were cut horizontally into two strips. The GFAP and β-actin were incubated with the same membrane. Primary antibodies were as follows: phosphorylated IκBα (Cat. no. 2859, Cell Signaling Technology, Boston, MA, USA, dilution 1:1000), total IκBα (Cat. no. 4814, Cell Signaling Technology, Boston, MA, USA, dilution 1:1000), phosphorylated NF-κB (Cat. no. 3033, Cell Signaling Technology, Boston, MA, USA, dilution 1:1000), total NF-κB (Cat. no. 8242, Cell Signaling Technology, Boston, MA, USA, dilution 1:1000), β-actin (Cat. no. A2228, Sigma-Aldrich, St. Louis, MO, USA; dilution 1:20,000), and GFAP (Cat. no. MAB360, Millipore, Billerica, MA, USA; dilution 1:1000). Subsequently, the membrane was washed three times, and then the membrane was incubated with secondary antibodies at room temperature for 1h. Target bands were revealed with ECL detection kit (Bio-Rad, Hercules, CA, USA). Western blots were analyzed using Image J and GraphPad Prism 8.0 software to quantify the bands.

#### 4.3.5. Quantitative PCR

The RNA of hippocampus and cell was extracted with Trizol reagent (Invitrogen, Carlsbad, CA, USA). Reverse transcription was performed using Vazyme reverse transcription kit (Cat. no. R333-01, Vazyme Biotech Co., Ltd., Beijing, China). The cDNA was then amplified using SYBR qPCR Master Mix with a RT-qPCR machine (Bio-Rad). Primer sequences are shown in Table 1.

#### 4.3.6. Enzyme-Linked Immunosorbent Assay

Blood samples were left at room temperature for 2 h and centrifuged at 4 °C, 3000 rpm for 15 min to collect the supernatant. Serum levels of TNF-α (Cat. no. MTA00B, R&D Systems, Minneapolis, MN, USA), IL-1β (Cat. no. MLB00C, R&D Systems, Minneapolis, MN, USA), and IL-6 (Cat. no. M6000, R&D Systems, Minneapolis, MN, USA) were detected using Quantikine ELISA kits according to the manufacturer’s instructions.

### 4.4. Data Analysis

All data were analyzed using GraphPad Prism 8.0 software. The data are displayed as the arithmetic mean ± SEM. Groups above two were compared using two-way analysis of variance (ANOVA) followed by Turkey’s post hoc tests. Any *p*-values less than 0.05 were considered statistically significant for all tests.

## 5. Conclusions

In the present study, we demonstrated that GP-14 extracted from gypenosides reduced neuroinflammation by inhibiting astrocytes activation via downregulation of NF-κB signaling pathway in a LPS-induced mice model and then improved depression-like behavior (as shown in Figure 8). Those results indicated for the first time that GP-14 could be a novel therapeutic approach for the treatment of depression and has a potential application for inflammatory-related diseases.

## Figures and Tables

**Table 1:** QPCR primer sequences.

Genes	Sequences
IL-6	forward:5′-ACTGTCGAGTCGCGTCCA-3′ reverse:5′-GTCATCCATGGCGAACTGGT-3′
IL-1β	forward:5′-TTCAGGCAGGCAGTATCACTC-3′ reverse: 5′-GAAGGTCCACGGGAAAGACAC-3′
TNF-α	forward: 5′-CCCTCACACTCAGATCATCTTCT-3′ reverse: 5′-GCTACGACGTGGGCTACAG-3′
β-actin	forward: 5′-ACTGTCGAGTCGCGTCCA-3′ reverse: 5′-GTCATCCATGGCGAACTGGT-3′

## Data Availability

Data are contained within the article.

## Supplementary Materials

The following supporting information can be downloaded at: https://www.mdpi.com/article/10.3390/ph16081152/s1. Figure S1: The effect of GP-14 on inflammatory factors in hippocampal tissue. (A) ELISA assay for the expression of IL-6 in hippocampal tissue. (B) ELISA assay for the expression of IL-1β in hippocampal tissue. The results were presented as mean ± SEM (*n* = 8/group). ** *p* < 0.01.
