# Supplementary material for: Gypenoside-14 Reduces Depression via Downregulation of the Nuclear Factor Kappa B (NF-kB) Signaling Pathway on the Lipopolysaccharide (LPS)-Induced Depression Model
Source: Pharmaceuticals (Basel). 2023 Aug 14;16(8):1152. doi: 10.3390/ph16081152 (PMC10459727; doi:10.3390/ph16081152)
Supplement: Supplementary file 1 [file pharmaceuticals-16-01152-s001.zip › pharmaceuticals-2523524-supplementary.pdf]

Supplementary materials

Gypenoside-14 Reduces Depression via Downregulation of the Nuclear Factor Kappa B (NF-kB) Signaling Pathway on the Lipopolysaccharide (LPS)-Induced Depression Model

Yaqun Jiang [1,†], Xiang Cheng [2,†], Ming Zhao [2], Tong Zhao [2], Mengya Zhang [1], Zibi Shi [2], Xiangpei Yue [2], Yanan Geng [2], Jiayue Gao [2], Chengbo Wang [3], Junli Yang [3,*], and Lingling Zhu [1,2,3,4,*]

[1] School of Pharmaceutical Sciences, University of South China, Hengyang 421001, China; yaqunjiang2023@163.com (Y.J.); zhmyasz@163.com (M.Z.)
[2] Beijing Institute of Basic Medical Sciences, Beijing 100850, China; mac_chx@163.com (X.C.); mingzhao28081@gmail.com (M.Z.); 13661091893@163.com (T.Z.); zibishi1994@163.com (Z.S.); xiangpei2015@sina.com (X.Y.); gengtangrong@163.com (Y.G.); gaojy0228@163.com (J.G.)
[3] CAS Key Laboratory of Chemistry of Northwestern Plant Resources and Key Laboratory for Natural Medicine of Gansu Province, Lanzhou Institute of Chemical Physics (LICP), Chinese Academy of Sciences (CAS), Lanzhou 730000, China; wangcb@licp.cas.cn
[4] Co-Innovation Center of Neuroregeneration, Nantong University, Nantong 226019, China

* Correspondence: yangjl@licp.cas.cn (J.Y.); linglingzhuamms@126.com (L.Z.)
† These authors contributed equally to this work.

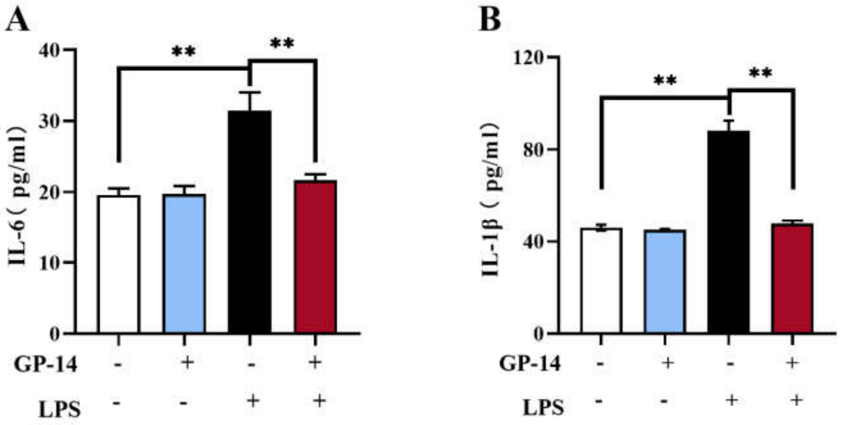

Figure S1: The effect of GP-14 on inflammatory factors in hippocampal tissue. (**A**) ELISA assay for the expression of IL-6 in hippocampal tissue. (**B**) ELISA assay for the expression of IL-1β in hippocampal tissue. The results were presented as mean ± SEM (*n* = 8/group). ** $p < 0.01$.
